# Hypoxia and Fatigue Impair Rapid Torque Development of Knee Extensors in Elite Alpine Skiers

**DOI:** 10.3389/fphys.2018.00962

**Published:** 2018-07-25

**Authors:** Marine Alhammoud, Baptiste Morel, Olivier Girard, Sebastien Racinais, Violaine Sevrez, Alexandre Germain, Thomas Chamu, Christophe Hautier

**Affiliations:** ^1^Inter-university Laboratory of Human Movement Biology (EA 7424), Claude Bernard University Lyon 1, Lyon, France; ^2^French Ski Federation, Annecy, France; ^3^Laboratory “Movement, Interactions, Performance” (EA 4334), Le Mans University, Le Mans, France; ^4^School of Psychology and Exercise Science, Murdoch University, Perth, WA, Australia; ^5^Athlete Health and Performance Research Centre, Aspetar Orthopedic and Sports Medicine Hospital, Doha, Qatar; ^6^Laboratory Sport, Expertise and Performance, French Institute of Sport (INSEP), Paris, France; ^7^French National Center for Scientific Research, Institute of Movement Sciences, Aix-Marseille University, Marseille, France; ^8^Orthopedic and Sports Medicine Hospital La Sauvegarde, Lyon, France

**Keywords:** isokinetic, maximal torque production, near-infrared spectroscopy, neural drive, repeated knee extensions, simulated altitude

## Abstract

This study examined the effects of acute hypoxia on maximal and explosive torque and fatigability in knee extensors of skiers. Twenty-two elite male alpine skiers performed 35 maximal, repeated isokinetic knee extensions at 180°s^-1^ (total exercise duration 61.25 s) in normoxia (NOR, FiO_2_ 0.21) and normobaric hypoxia (HYP, FiO_2_ 0.13) in a randomized, single-blind design. Peak torque and rate of torque development (RTD) from 0 to 100 ms and associated *Vastus Lateralis* peak EMG activity and rate of EMG rise (RER) were determined for each contraction. Relative changes in deoxyhemoglobin concentration of the VL muscle were monitored by near-infrared spectroscopy. Peak torque and peak EMG activity did not differ between conditions and decreased similarly with fatigue (*p* < 0.001), with peak torque decreasing continuously but EMG activity decreasing significantly after 30 contractions only. Compared to NOR, RTD, and RER values were lower in HYP during the first 12 and 9 contractions, respectively (both *p* < 0.05). Deoxyhemoglobin concentration during the last five contractions was higher in HYP than NOR (*p* = 0.050) but the delta between maximal and minimal deoxyhemoglobin for each contraction was similar in HYP and NOR suggesting a similar muscle O_2_ utilization. Post-exercise heart rate (138 ± 24 bpm) and blood lactate concentration (5.8 ± 3.1 mmol.l^-1^) did not differ between conditions. Arterial oxygen saturation was significantly lower (84 ± 4 vs. 98 ± 1%, *p* < 0.001) and ratings of perceived exertion higher (6 ± 1 vs. 5 ± 1, *p* < 0.001) in HYP than NOR. In summary, hypoxia limits RTD via a decrease in neural drive in elite alpine skiers undertaking maximal repeated isokinetic knee extensions, but the effect of hypoxic exposure is negated as fatigue develops. Isokinetic testing protocols for elite alpine skiers should incorporate RTD and RER measurements as they display a higher sensitivity than peak torque and EMG activity.

## Introduction

Alpine skiing requires a high activation level of the knee extensor muscles to sustain repeated, near maximal contractions (Ferguson, 2010) for 45–120 s (Berg and Eiken, 1999). Exercise-induced muscle fatigue can be defined as a reduction in the maximum force that a muscle can exert and/or sustain (Enoka, 2002). Reportedly, skiing-induced fatigue is manifested by decreases in hamstring and quadriceps eccentric torque for 1–24 h following a 4-h skiing session (Koller et al., 2015). Hence, skiing-induced fatigue alters force production capacity and electromyographic (EMG) activity in the lower extremities (Kröll et al., 2005, 2011; Ushiyama et al., 2005; Akutsu et al., 2008; Tomazin et al., 2008; Kiryu et al., 2011). Neuromuscular consequences of fatigue development for the *vastus lateralis* (VL) muscle also include a decline in mean power frequency in the first half of a 1–2 min ski run (Ushiyama et al., 2005) and the presence of high-frequency fatigue after ∼45 s of slalom (Tomazin et al., 2008).

Recent studies have suggested that the rate of torque development (RTD) within the initial phase of a contraction represents a more functional outcome measure than maximal torque *per se* (Girard and Millet, 2009; Jordan et al., 2015). A decreased in RTD was also associated with poor jumping/hopping performance and abnormal knee loading, potentially increasing injury risk (Pua et al., 2017). RTD is important to stabilize the musculo-skeletal system in response to mechanical perturbation (Folland et al., 2014). This may especially be true in skiing where the time available to develop force is short. Arguably, a high RTD may help preventing falls and injury in alpine skiing, where anterior cruciate ligament (ACL) injury typically occurs in a time-window of 200 ms (Bere et al., 2011). Indeed, alpine skiing requires immediate postural adjustments to prevent falls in response to a loss of balance onto uneven ski slopes at high speed. Importantly, a greater injury risk was reported in World Cup skiers in the final section of the course pointing out fatigue as a possible contributing factor (Bere et al., 2014). Thus, studying the effect of fatigue on RTD may help to shed more light on the underpinning neuromuscular factors responsible for the high injury rate in elite skiers (Jordan et al., 2015, 2017; Haaland et al., 2016).

The repetition of near maximal contractions while skiing also generate high intramuscular pressures, likely reducing muscle perfusion and thereby oxygen delivery/waste removal (Szmedra et al., 2001). This effect may be more prominent in Giant Slalom (GS) than Slalom (SL) due to a more flex position with higher forces (Turnbull et al., 2009), and even more pronounced in Super Giant Slalom where the average knee angular velocity is low (Berg and Eiken, 1999). Using near-infrared spectroscopy (NIRS) a 33% greater oxygen desaturation of the VL along with a 30% greater blood volume change have been reported during GS than SL (Szmedra et al., 2001). In competitive speed-skating, increased deoxygenation in the capillary bed of the exercising quadriceps was demonstrated during characteristic low sitting versus upright position (Rundell et al., 1997). Given that the rhythmic pattern of GS or SL only allows a partial reperfusion of working muscles in alpine skiers (Szmedra et al., 2001), a comprehensive investigation of the time course and etiology of fatigue development in skiers should also include quadriceps muscle deoxygenation trends.

Lastly, the vascular occlusions during the forceful contractions of ski racing (Tesch et al., 1978) increase lactate production, as does the hypoxic environment when competing at altitude (Turnbull et al., 2009). Indeed, alpine skiing is performed at moderate-to-high terrestrial altitude, with sometimes starting gate as high as ∼3,500 m above sea level (Beaver Creek, CO, United States), creating a unique challenge on skiers. Hypoxia has been demonstrated to impair exercise capacity during repeated, maximal leg extensions (Goodall et al., 2010; Christian et al., 2014) partly through an alteration in neural drive (Amann and Calbet, 2008; Goodall et al., 2010; Billaut et al., 2013). Such alterations, when altitude severity is >2000 m, can increase risk of fall and injury occurrence in recreational skiers (Burtscher et al., 2009). The acute effects of hypoxia on performance involving repeated bouts of short high intensity efforts without long recovery time are not negligible and deserve more research attention in elite skiers (Chapman et al., 2010).

The aim of this study was to assess the time course and magnitude of changes in peak and rapid torque production together with neuromuscular and metabolic adjustments in the VL during repeated maximal leg extensions in elite alpine skiers. We hypothesized that, compared to normoxia, hypoxia exposure would exacerbate muscle deoxygenation causing further reductions in maximal torque and rapid torque development through a lower central motor drive.

## Materials and Methods

### Participants

Twenty-two elite male alpine skiers (mean ± SD stature 179 ± 4 cm, body mass 83 ± 6 kg, age 26 ± 4 years) participated in this study. All were members of the French national team (including world champions and Olympic medalists) with a background training of >200 days per year for several years. The project was approved by the Aspetar scientific committee (approval number CMO/000058/fj) and the ADL-Q ethics committee (approval number E20140000011). All subjects gave written informed consent in accordance with the Declaration of Helsinki.

### Experimental Design

Participants performed a fatiguing exercise (see below) in normoxic [NOR, sea level, inspired O_2_ fraction (FiO_2_) 0.21] and normobaric hypoxic (HYP, simulated altitude of 3,800 m, FiO_2_ 0.13) conditions, in a random order and using a single-blind design. The two exercise tests took place on the same day and were separated by 2 h, during which the participants resting quietly on a chair with no specific recovery intervention. This was based on the observation that 10–30-min rest periods allow an almost complete recovery of neuromuscular function parameters after high-intensity single-joint quadriceps exercise (Gruet et al., 2014) or repeated sprint running exercise (Perrey et al., 2010). Additionally, the elite athletes recruited here were used to train twice a day and were accustomed to heavy resistance training. This procedure also permitted to keep the EMG sensors at the exact same location to avoid methodological difficulty in comparing EMG data on separate days. All participants were tested between 9:00 and 19:00 in a temperate room, with no other training done on that day. Participants were instructed to avoid strenuous activities for 48 h before testing and to follow their normal diet and sleeping habits. Prior to the fatiguing exercise, participants completed a standardized warm-up including 10 min of cycling at 1 W⋅kg^-1^ followed by knee extensions (8 × 240°s^-1^; 6 × 180°s^-1^; 4 × 90°s^-1^; 2 × 30°s^-1^; 2 × 0°s^-1^; with 45 s recovery between sets) at progressively increased subjective awareness of effort (70–100% of maximal perceived intensity). Thereafter, participants rested in a seated position for 5 min before exercise commencement. For both trials, a facemask connected to a portable hypoxic generator (Altitrainer, SMTEC, Nyon, Switzerland), controlling the FiO_2_ at the required level during the warm-up knee extensions and the fatiguing exercise, was attached on subjects. All participants were used to the system as they regularly use it during their training. However, they were not specifically acclimated to hypoxia when experiments were conducted as testing occurred during the early pre-season (June), approximately 1 month before engaging in a complete block of altitude training (July–August).

### Exercise

The fatigue protocol was performed on an isokinetic dynamometer (Contrex, CMV AG, Dübendorf, Switzerland). Calculation of the limb weight was carried out during passive movements and gravity correction was performed with the ConTrex-MJ software (Contrex, CMV AG, Dübendorf, Switzerland). The subjects were seated with their hip joint angles set at 80° (0° is full extension) and their chest and working leg tightly fixed against the chair. They were asked to cross their arms over the chest during the contractions. The protocol consisted of 35 maximal, repeated isokinetic knee extensions of the dominant leg at 180°⋅s^-1^, from 90° to 45° of knee flexion (0° is full knee extension). Each contraction lasted 250 ms and the total exercise duration was 61.25 s. At the end of each maximal knee extension, the participants were instructed to relax their leg while the isokinetic device returned to its initial position at 30°s^-1^ (i.e., 1.5 s). Participants were instructed to “push as fast and as hard as possible.” While the protocol involves the completion of 35 knee extensions only the first 34 were further analyzed to avoid the bias of the apparatus stop. Participants were provided with a real-time feedback allowing them to visualize the torque produced during each maximal knee extension. Strong verbal encouragement was provided throughout.

### Torque Measurements

Torque and angular velocity were recorded (sampling rate: 256 Hz) to calculate peak torque (T_peak_) and RTD. RTD was calculated as the slope of the torque vs. time curve between 0 and 100 ms relative to the contraction onset. We have decided to focus our analysis on the initial 100 ms after contraction onset for the following reasons: (1) RTD seems to be mainly determined by the capacity to produce maximal voluntary activation in the early phase of an explosive contraction (within 100 ms) particularly as a result of increased motor unit discharge rate; (2) there is a greater variability during the early phase of the contraction (especially the first 50 ms); (3) despite rapid muscle activation being a critical determinant of RTD, the voluntary RTD becomes increasingly influenced by muscle properties and maximal force for duration longer than 100 ms (Maffiuletti et al., 2016).

### Electromyography

EMG activity of the VL was recorded using surface electrodes (EMG Triode, nickel-plated brass, electrode diameter = 1 cm, inter-electrode distance = 2 cm, Thought Technology, Montreal, QC, Canada). The same electrodes were kept in place for both tests sessions to ensure that EMG activity signals representative of the same muscle area could be compared by using repeated measures. Albeit surface EMG recordings can be affected by extreme environmental temperatures (Bell, 1993; Racinais, 2013), the current protocol was performed under temperate ambient conditions. EMG signals were sampled at 2048 Hz using the Flexcomp Infiniti system (Thought Technology, Montreal, Canada). The system had an input impedance and common mode rejection ratio of 2 MΩ and >110 dB, respectively. The skin was shaved and cleaned with alcohol before placing electrodes to improve the contact between skin and electrode and to reduce skin impedance. Raw EMGs were filtered (Butterworth order 2, bandpass 10-500 Hz) and amplified (gain = 500) before calculating root mean squared values (RMS) with a 50-ms moving rectangular window (MATLAB scripts, Mathworks, Natick, MA). The onset of the RMS bursts was detected using the threshold method described by Morel et al. (2015) based on ± 3 SD of the resting baseline. For each contraction, the following EMG variables were extracted: Peak EMG (EMG_peak_) calculated over a 50 ms period and RER defined as the slope of the EMG–time curve between 0 and 100 ms relative to the activation onset.

### Muscle Oxygenation Trends

**FIGURE 1:**
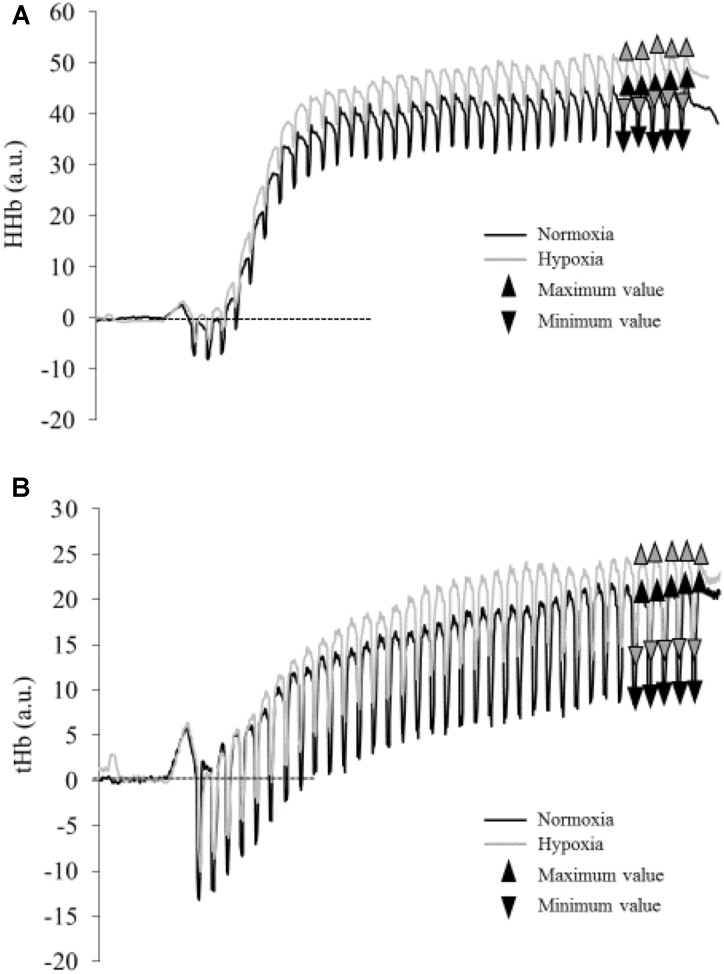
Typical traces of deoxyhemoglobin concentration [HHb] **(A)** and total hemoglobin (tHb) **(B)** during the fatiguing protocol in normoxia (black line) and hypoxia (gray line) for one subject. Triangles represent maximal and minimal values measured during the last five contractions (mean values are presented in **Table 1**).

Relative changes in deoxyhemoglobin concentration [HHb] of the VL muscle were monitored (sampling frequency 20 Hz) by NIRS (Portamon, Artinis, Zetten, Netherlands). Whereas various parameters can be extracted by NIRS, we selected [HHb] as it is insensitive to blood volume during exercise (De Blasi et al., 1993; Ferrari et al., 1997), and thus represents a reliable indicator of changes in oxygen extraction when investigating exercise-induced fatigue (De Blasi et al., 1994; Ferrari et al., 1997). The optodes were fixed at 40 mm distance between the light source and the detector. The optode assembly was secured on the cleaned skin surface with tape and then covered with a black cotton tissue. The light emitted by the infrared probe is assumed to reach a tissue depth of 50% of the interoptode spacing (Matsushita et al., 1998). Skinfold thickness was measured using a skinfold caliper. The obtained value was 6.5 ± 2.9 mm, which was well below the penetration depth of the NIRS photons. A differential pathlength factor of 3.8 was used for the VL muscle (DeLorey et al., 2005). Changes in [HHb] were reported as an absolute change from baseline that was measured during 1 min at rest before the exercise and were used as an estimator of changes in intramuscular oxygenation. Total hemoglobin (tHb) was also calculated. Changes in [HHb] and tHb during the exercise were analyzed during the last 5 contractions (i.e., at steady state) as the mean difference between maximal and minimal values (Δ min-max [HHb], Δ min-max tHb, respectively) (**Figure 1**).

### Systemic Responses

#### Heart Rate

Heart rate (HR) was continuously monitored at 5-s intervals during all trials using a heart rate monitor connected to a chest strap (RS800, Polar Electro OY, Kempele, Finland) and the resting and post-exercise (i.e., 15 s after exercise cessation) values were extracted.

#### Ratings of Perceived Exertion

After each session, participants were asked to indicate their ratings of perceived exertion (RPE) using a Borg CR-10 scale (Borg, 1990).

#### Arterial Saturation

Percent arterial oxygen saturation (SpO_2_, %) was measured before and at the end of the exercise using a fingertip pulse oximeter (Onyx II, Model 9560, Nonin, Plymouth, MN, United States).

#### Blood Lactate

A micro blood sample was taken from the fingertip before and 3 min after the end of the exercise. The sample was analyzed for lactate concentration using a Lactate Pro (LT-1710, Arkray, Japan) portable analyzer.

### Statistical Analysis

Data were analyzed with Statistica 8.0 Software (Stat Soft Inc.^®^, Tulsa, OK, United States). The normality of the error distribution was examined with Lilliefors’ test. Homogeneity of variance was verified using Levene’s test. With the assumption of normality and homogeneity of variance confirmed, data were analyzed using two-ways repeated-measures ANOVAs (time x condition). Tukey’s *post hoc* tests were used. Finally, a paired Student’s *t*-test was performed to compare the blood lactate accumulation, SpO_2_, end exercise heart rate, and rate of perceived exertion during NOR and HYP. Effect-sizes are described in terms of partial eta-squared (η^2^, with η^2^≥ 0.06 representing a moderate effect and η^2^≥ 0.14 a large effect). Alpha level for statistical significance was set at *p* ≤ 0.05. Data are presented as means ± Standard Deviation (SD). 95% confidence interval (CI95%) are reported for [HHb] and tHb data.

## Results

### Torque

**FIGURE 2:**
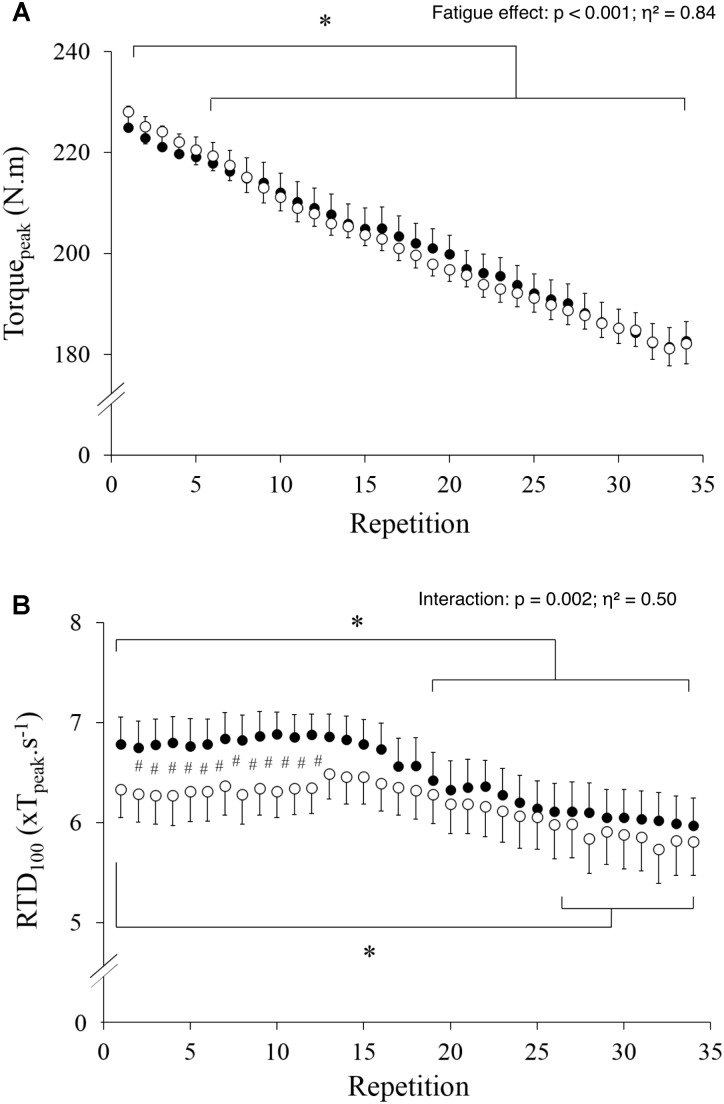
Maximal torque **(A)** and rate of torque development **(B)** values measured during the 34 maximal contractions performed in normoxia (black dots) and hypoxia (open circle). Data are means ± SD for 22 participants. #*p* < 0.05 hypoxia versus normoxia; ^∗^*p* < 0.001 effect of fatigue.

T_peak_ decreased steadily during the exercise (**Figure 2A**, *p* < 0.001; η^2^= 0.84), without a significant difference between conditions (*p* = 0.992) or any interaction effect (*p* = 0.725). Conversely, there was an interaction effect on RTD (**Figure 2B**, *p* = 0.002; η^2^= 0.50) with significantly lower values in HYP vs. NOR during the first 12 contractions only. Compared to the first contraction, RTD values decreased significantly from the nineteenth contraction in NOR and from the twenty-eighth contraction in HYP.

### EMG

**FIGURE 3:**
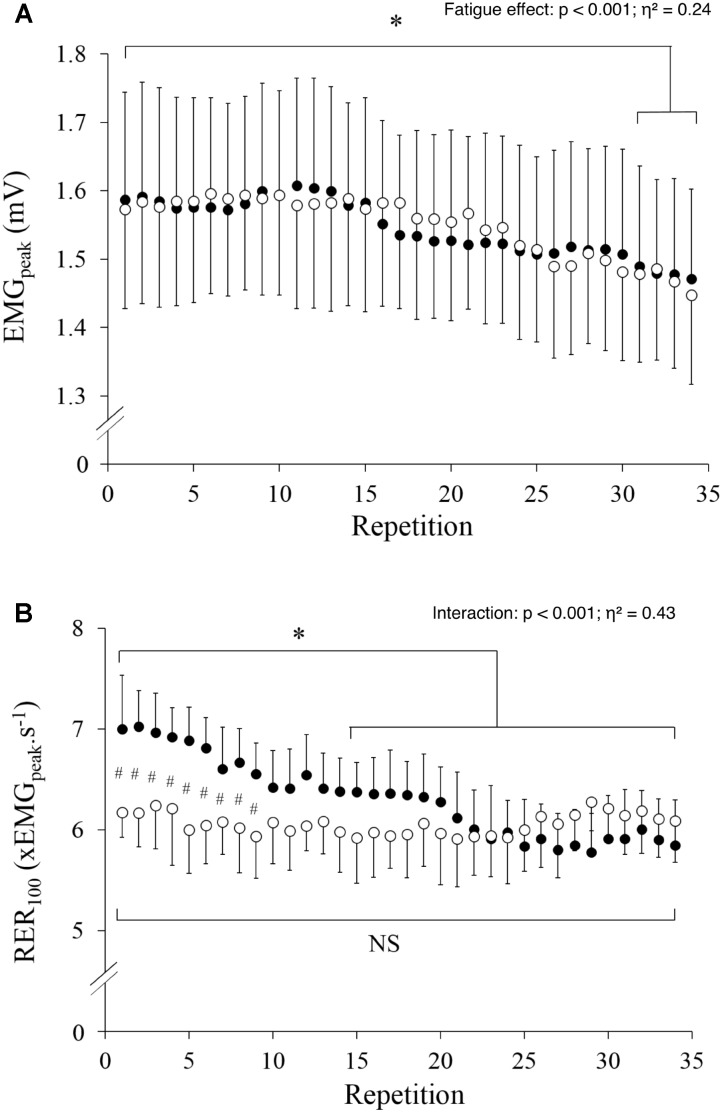
Peak EMG **(A)** and rate of EMG rise **(B)** measured during the 34 maximal contractions performed in normoxia (black dots) and hypoxia (open circle). Data are means ± SD for 22 participants. #*p* < 0.05 hypoxia versus normoxia; ^∗^*p* < 0.001 effect of fatigue.

EMG_peak_ significantly decreased with fatigue (*p* < 0.001; η^2^= 0.24) and was lower during the last four contractions as compared to the first contraction (**Figure 3A**), yet without a significant difference between conditions (*p* = 0.951) or any interaction effect (*p* = 0.324). Conversely, there was an interaction effect on RER (**Figure 3B**, *p* < 0.001; η^2^= 0.43) with significantly lower values in HYP vs. NOR during the first 9 contractions only. RER significantly decreased with fatigue in NOR, whereas it remained unchanged in HYP (**Figure 3B**).

### Muscle Deoxygenation

The maximal [HHb] (*p* = 0.050; η^2^= 0.25) but not tHb (*p* = 0.186; η^2^= 0.12) measured during the last 5 contractions was higher in HYP compared to NOR (**Table 1** and **Figure 1**). However, the delta between maximal and minimal [HHb] and tHb throughout the last five contractions (**Table 1**) did not differ between conditions (Δ[HHb] *p* = 0.956, ΔtHb *p* = 0.252).

**Table 1:** Deoxyhemoglobin [HHb] and total hemoglobin (tHb) averaged over the last five contractions in normoxia and hypoxia.

	[HHb]		tHb	
	*Normoxia*	*Hypoxia*	*Normoxia*	*Hypoxia*
Peak value (a.u.)	38.9 ± 15.7	41.0 ± 12.8^∗^	17.5 ± 8.6	15.8 ± 8.1
CI95% peak	*32.3–45.4*	*35.6–46.3*	*13.9–21.1*	*12.4–19.2*
Δmin-max (a.u.)	10.1 ± 9.8	11.0 ± 5.5	14.6 ± 8.7	13.9 ± 7.2
CI95% Δmin-max	*6.0–14.2*	*8.7–13.2*	*10.9–18.2*	*10.9–16.9*


^∗^*p* ≤ 0.05.

### Systemic Responses

HR (77 ± 16 vs. 138 ± 24 bpm; *p* < 0.001; η^2^= 0.90) and lactate (2.5 ± 1.4 vs. 5.8 ± 3.1 mmol.l^-1^; *p* < 0.001; η^2^= 0.72) increased from pre to post-exercise (both *p* < 0.001), independently of condition (HR *p* = 0.890, lactate *p* = 0.630). A significant interaction between time and condition was revealed for SpO_2_ (*p* < 0.001; η^2^= 0.81); SpO_2_ values decreased from pre to post-exercise in HYP (92 ± 2% vs. 84 ± 4%; *p* < 0.001), whereas it did not change in NOR (98 ± 2% vs. 98 ± 1%; *p* = 0.99). RPE values were higher in HYP (5.9 ± 2.8) vs. NOR (5.1 ± 1.1) (*p* < 0.001; η^2^ = 0.56).

## Discussion

The objective of the present study was to determine the effects of acute hypoxic exposure on alterations in maximal and rapid torque production and accompanying neuromuscular and metabolic adjustments during maximal, repeated isokinetic knee extensions in elite alpine skiers. Our main findings were that hypoxia caused reductions in RTD and RER, whereas T_peak_ and EMG_peak_ remained unchanged. Importantly, the subsequent exercise-induced decreases in RTD and RER were of smaller magnitude when exercising under hypoxia, leading to similar end-exercise values with also matched muscle de-oxygenation levels between conditions.

### T_peak_ and EMG_peak_

In line with previous studies (Kawahara et al., 2008; Goodall et al., 2012; Christian et al., 2014), hypoxia exposure *per se* had no effect on maximal torque during a single contraction (**Figure 2A**). However, the effect of hypoxia on fatigue when contractions are repeated is more contentious in the literature with some studies reporting larger decline in torque in HYP than NOR (Goodall et al., 2010; Christian et al., 2014), whereas other concluded to no differences (Kawahara et al., 2008). Our current results confirmed the later, with a similar decrement in T_peak_ induced by fatigue in HYP and NOR. However, with only one single severity of altitude simulation tested, it remains to be verified if graded hypoxia (with lower or more severe hypoxic conditions) actually modifies the extent of maximal force and EMG responses to our exhaustive exercise. Interestingly, the magnitude and nature (contribution of neural vs. peripheral factors) of fatigue-induced adjustments in neuromuscular function after unilateral knee extensions is dependent of hypoxia severity (Goodall et al., 2010). This implies that interpretation of our results remains specific to FiO_2_ 0.13 (3,800 m), acknowledging that there is currently no alpine ski race starting at a higher altitude. The current results may also be specific to the population tested who is possibly less vulnerable to fatigue. Indeed, the skiers participating in this study routinely trained at altitude and elite athletes are likely to have a higher capillary density and capillary-to-fiber ratio than untrained participants (Zoladz et al., 2005); fatigue and recovery has been correlated with capillary density (Tesch and Wright, 1983) in the VL after 50 repeated consecutive maximal voluntary contractions at 180°s^-1^ but not with fiber type distribution.

As for T_peak_, our data show an effect of fatigue on EMG_peak_ but no effect of hypoxia (**Figure 3A**). This indicates that hypoxia didn’t exaggerate fatigue-induced decrease in maximal neural drive to the VL of elite skiers. This partly differs from a previous report suggesting that a diminished O_2_ availability in the brain could cause a failure of drive from the motor cortex during whole-body exercise (Goodall et al., 2012), with the downregulation in quadriceps muscle recruitment under hypoxia limiting muscle fatigue (Billaut et al., 2013). However, this is in line with the observation that hypoxia does not modify central regulation of motor drive during localized exercise involving a small muscle mass (Rupp et al., 2015). EMG_peak_ was maintained for the first 15 contractions in the current study despite an overall decrease in T_peak_ and a lower SpO_2_ in HYP, suggesting that the decrease in T_peak_ was mainly due to peripheral fatigue with no or limited recruitment downregulation during maximal contractions. This also complete previous reports showing that elite skiers better tolerate fatigue (Akutsu et al., 2008) or environmental conditions such as cold (Racinais et al., 2017) than the general population.

### RTD and RER

There has been a shift toward power training and heavier skiers over the past years in alpine ski racing (Turnbull et al., 2009) and top-level power athletes are characterized by a markedly greater knee extensor RTD measured during the initial 150 ms than habitually active individuals (Tillin et al., 2010). However, our results showed that RTD is reduced by hypoxia, i.e., a common environmental stressor for alpine skiers. The unique finding that hypoxia impairs RTD with a preserved T_peak_ might at first appear in contradiction with the further reduction in T_peak_, but not RTD, that was previously reported following repeated sprints in hypoxia (Girard et al., 2016). However, in that previous study, the neuromuscular test battery was performed in normoxia before and after the fatiguing task. When only considering end of exercise values, our results also failed to show any significant difference in RTD between HYP and NOR. In the current study, RTD was reduced in HYP compared to NOR during the first 12 contractions only (**Figure 2B**). According to Edman and Josephson (Edman and Josephson, 2007), RTD is influenced by passive stiffness, fiber type composition, cross-bridge kinetics and neural drive. Because the two first muscular factors were probably not modified under hypoxia at the beginning of the protocol, RTD decrease could possibly be explained by cross-bridge kinetics and neural drive. Cross-bridge kinetics is influenced by metabolites accumulation and it is unlikely that this parameter was different during the first few contractions between HYP and NOR. Thus, a lower neural drive, as demonstrated by the lower RER under hypoxia in the first contractions (**Figure 3B**), likely is the primarily cause of hypoxia-induced decrement in RTD. While it may appear counterintuitive that HYP affected RER during the first 9 contractions, it should be clarified that some physiological responses were already impacted by HYP from the beginning of the exercise (e.g., SpO2 was 92% in HYP vs. 98% in NOR). No changes in EMG_peak_ were observed with such rather narrow (yet clinically relevant) difference in SpO2, which may, however, have triggered a decrement in RER by teleoanticipation of the upcoming 61 s of exercise. In support, it was previously demonstrated that “all-out” pacing strategies are adopted for exercise up to 15 s, but pacing becomes apparent when exercise is expected to last 30 s or more despite the instruction to go “all-out” (Wittekind et al., 2011). This observation adds to a previous report that whereas T_peak_ alterations are related to peripheral perturbations, RTD losses were associated to both central and peripheral fatigue in non-athletes in normoxic environment (Morel et al., 2015).

It should be acknowledged that other factors (not investigated in the current protocol) are also likely to affect RTD while skiing. Indeed, skiing is generally performed in cold environments. Both cold and hypoxia exposure individually decrease constant-workload (high-intensity) knee extension time to exhaustion, with the decrements being additive when both stressors are combined (Lloyd et al., 2016). This decrease was attributed to a faster rate of peripheral fatigue development (Lloyd et al., 2016). Moreover, colder muscle temperature is also known to specifically decrease RTD (Zhou et al., 1998). Lastly, there is an ongoing debate on the potential physiological differences between hypobaric and normobaric hypoxia (Millet et al., 2012). However, this debate mainly concerns aerobic adaptations or acute mountain sickness rather than neuromuscular function adjustments (Millet et al., 2012). The only specificity of real altitude while skiing relates to a decrease in aerodynamic resistances, which remains challenging to replicate under controlled laboratory conditions.

### SpO_2_ and NIRS Data

As expected, hypoxia lowered SpO_2_ before and after the exercise, which occurred along with higher muscle [HHb] values (**Figures 1A,B**). It should, however, be acknowledged that muscle oxygen desaturation may be larger during skiing than during isokinetic testing due to low posture, prolonged near-maximal muscle contraction and a rhythmic pattern with insufficient time to adequately re-perfuse working muscle (Szmedra et al., 2001). This may affect blood flow and oxygen delivery to working muscle. Reportedly, changes in hemoglobin/myoglobin oxygen desaturation between rest and exercise, expressed relatively to maximal oxygen desaturation determined with cuff ischemia, are greater during GS (79.2%) than SL (65.7%) (Szmedra et al., 2001). Because our data were not normalized to maximal oxygen desaturation obtained with cuff ischemia, no comparison could be made for the relative desaturation observed in the current study with this previous literature. However, irrespectively, of the absolute levels, it is of interest to note that the delta between maximal and minimal [HHb] values calculated during the last contractions were identical in the two conditions, using repeated measures with the same methods, indicating that muscle O_2_ utilization was similar. In addition, the blood lactate accumulation was similar in NOR and HYP, suggesting an identical anaerobic energy production. These observations differ from Calbet et al. (2003) who proposed that aerobic ATP production is reduced under hypoxia and compensated by an enhanced anaerobic energy production during a Wingate test. However, the active muscle mass was relatively small in the present study compared to cycling sprint, and O_2_ delivery to the quadriceps muscle may thus be sufficient to maintain the aerobic energy supply even when oxygen availability is reduced. Thus, the similar O_2_ utilization and lactate production, with lower SpO_2_ and higher RPE in HYP, indicate similar muscle energy turnover but higher whole-body stress during single leg exercise under reduced O_2_ availability.

### Limitations

We selected the first 100 ms as the importance of a high RTD for superior sprint/acceleration capacity, in rugby players for instance, was strongly related to the proportion of maximal force achieved in the initial phase (i.e., only for RTD 100 ms) of explosive-isometric squats (Tillin et al., 2013). However, the relative contribution of the central and peripheral factors to RTD throughout the rising phase of the force-time curve may depend on the time intervals considered for analysis. A number of different windows (0–50, 50–100, 0–150, or 100–200 ms) have been used in previous studies (Jordan et al., 2015; Maffiuletti et al., 2016) and shorter (<100 ms) or longer (>100 ms) analyses may have shown slightly different results, shorter windows being more influenced by central factors (Maffiuletti et al., 2016).

In order to mimic alpine skiing, it would be necessary to sequentially decrease the severity of hypoxic exposure during the exercise as vertical drop in official competition typically ranges from 180 m (SL) to 1100 m (Downhill) within 2 min. In addition, the exercise-to-rest ratio together with the number of contractions of the fatiguing protocol could also be extended. Indeed, the gate number is 55–75 for a SL and above 30 gates for GS. Besides, the angular velocity chosen and contraction mode (concentric) may not be representative of alpine skiing discipline, even though an angular velocity of 180°/s has often been used in fatigue isokinetic protocols in footballers (Sangnier and Tourny-Chollet, 2008). Indeed, lower angular velocity (20–70°/s) with deeper knee flexion (67–140°) and predominance of eccentric/isometric work in a closed kinetic chain has been previously seen in alpine skiing (Berg and Eiken, 1999). However, RTD is typically measured under isometric conditions despite speed-related differences in motor unit activation pattern influencing rapid muscle force production (Maffiuletti et al., 2016). Occasionally, RTD has been measured during squat or leg-press which may be more relevant for practical outcomes (Maffiuletti et al., 2016). Thus, future studies should consider measuring RTD in skiers with press or during squat exercises and testing movement patterns that mimic the ski activity pattern. We acknowledge that our protocol may not be representative of alpine skiing exercise for the above-mentioned reasons, nonetheless we choose it as it allowed assessing RTD under controlled conditions.

### Practical Applications

Recent reviews point out the critical need to develop ski-specific neuromuscular screening tests and prevention programs (Jordan et al., 2017). Our results seem to support the notion that isokinetic testing protocols for elite skiers should incorporate RTD measurements. A more specific testing should also incorporate an evaluation of fatigue and hypoxia effects to better understand the skier neuromuscular function in a practical context of performing a ski run at altitude.

Because of the tremendous strength demands of ski racing especially on the quadriceps (Abe et al., 1992), the implementation of regular hamstring and quadriceps strength assessments with special reference to RTD seems warranted in the physical evaluation of uninjured skiers. In addition, Jordan et al. (2015) observed quadriceps maximal torque and RTD deficits in the ACL-reconstructed limb of the skiers up to 25 months after the surgery, whereas skiers received medical clearance to return to full competition. These deficits led to an inflated hamstring / quadriceps ratio compared with that in uninjured controls. Considering the high ACL injury and re-injury rate in elite skiers (Pujol et al., 2007), hamstring and quadriceps strength should be assessed over a long-term period after surgery to identify chronic strength deficits in ACL-reconstructed skiers. In summary, developing higher RTD values may help preventing falls and injury in alpine skiing, where ACL injury typically occurs within the first 200 ms of muscle contraction (Bere et al., 2011). Practically, RTD can be improved both by heavy-resistance and explosive strength training through hypertrophic and neural adaptations (Aagaard et al., 2002; Maffiuletti et al., 2016), which is already included in strength and conditioning program in elite skiers.

Resistance training in hypoxia (either systemic or with blood flow restriction) is a novel and popular training method potentially causing greater muscular development and strength gains versus similar training at sea level (Heitkamp, 2015; Inness et al., 2016) and a decreased fatigue in repeated sprints (Ramos-Campo et al., 2018a). Nevertheless, a recent meta-analysis concluded that resistance training in hypoxia did not produce significant change in muscular size or maximal strength when compared to normoxic resistance training (Ramos-Campo et al., 2018b). These strength gains concern usually maximal voluntary contraction but remain unclear for RTD as hypoxia-mediated neural adaptations have not yet been discovered. Further studies are therefore needed to evaluate the effectiveness of training using repeated maximal explosive contractions under simulated altitude as well as to document the dose-response relationship. We would expect an improvement in fatigue resistance on RTD (lesser drop), which is often considered a more sensitive parameter than maximal torque.

## Conclusion

Whereas fatigue-induced decrements in peak torque and EMG were similar in normoxia and hypoxia, the RTD and RER were initially lower during repeated, maximal isokinetic contractions in elite alpine skiers when oxygen availability was reduced. This suggests that hypoxia limits RTD via decreased neural drive to active musculature, but this effect is negated by fatigue as muscle oxygenation trends as well as RTD and RER decrements were of smaller magnitude under hypoxia. As such, muscle function integrity may be first negatively impacted by hypoxia exposure before fatigue mediated its effect on rapid torque production during alpine ski races.

## Data Availability

The raw data supporting the conclusions of this manuscript will be made available by the authors, without undue reservation, to any qualified researcher.

## Author Contributions

MA, SR, and CH designed the study. MA, BM, SR, VS, AG, TC, and CH collected the data. MA, BM, OG, SR, and CH analyzed the data and drafted the manuscript. All authors revised and approved the manuscript.

## Conflict of Interest Statement

The authors declare that the research was conducted in the absence of any commercial or financial relationships that could be construed as a potential conflict of interest.

## Abbreviations

EMGpeak: peak EMG
HYP: normobaric hypoxia
NOR: normoxia
RER: rate of EMG rise
Tpeak: peak torque
